# Treatment of Enteropathogenic *Escherichia coli* Diarrhea in Cancer Patients: A Series of Three Cases

**DOI:** 10.1155/2018/8438701

**Published:** 2018-04-19

**Authors:** James D. Denham, Sowmya Nanjappa, John N. Greene

**Affiliations:** ^1^University of South Florida Morsani College of Medicine, 12901 Bruce B. Downs Blvd., Tampa, FL 33612, USA; ^2^Department of Internal Medicine and Oncologic Sciences, H. Lee Moffitt Cancer Center, University of South Florida Morsani College of Medicine, 12902 Magnolia Drive, MCC-IHM, Tampa, FL 33612-9497, USA; ^3^Infectious Diseases and Hospital Epidemiology, Moffitt Cancer Center and Research Institute, 12902 Magnolia Drive, FOB-3, Tampa, FL 33612-9497, USA

## Abstract

Enteropathogenic *Escherichia coli* (EPEC) is a common cause of watery diarrhea in children in the developing world and an infrequent cause of significant diarrhea in adult patients. EPEC diarrhea, while not commonly seen in cancer patients, can cause significant distress to patients, and antimicrobial choice for this condition in this patient population is not clearly delineated in the literature. We report 3 cases of EPEC diarrhea in cancer patients and discuss the use of azithromycin for successful treatment of these patients. Positive outcomes were seen while using azithromycin in our first two patients and ciprofloxacin in our third patient.

## 1. Introduction

*Escherichia coli* is the prototypical coliform bacterium: a Gram-negative, facultatively anaerobic, lactose-fermenting rod. Enteropathogenic *Escherichia coli* (EPEC) is a non-Shiga toxin-producing strain of *E. coli* that causes diarrhea via an “attaching and effacing” mechanism on the surface of enterocytes [1, 2]. EPEC is traditionally associated with causing severe (and often fatal) watery diarrhea in infants in the developing world as well as in adults who travel to regions where bacterial diarrhea is endemic. In adults, EPEC diarrhea presents as watery diarrhea (sometimes associated with vomiting) in association with a low-grade fever. This illness, if left untreated, may persist for up to 120 days [3]. We report 3 cases of EPEC diarrhea in adult cancer patients at an academic cancer hospital and discuss the antimicrobial drugs used to treat these patients.

## 2. Case Series

### 2.1. Case 1

The first patient was a 55-year-old female with multiple myeloma (status-post chemotherapy, autologous hematopoietic stem cell transplantation, and radiotherapy). The patient was also enrolled in an experimental trial of antimyeloma therapy after it was determined that her multiple myeloma was progressing. Two days before presentation, she developed watery diarrhea. She reported that she had recently visited with her two grandchildren, ages 2 and 3, but they did not appear ill. The next day, she developed fevers as high as 103°F (39.4°C) associated with chills. The physical exam revealed no abnormalities. Laboratory studies ordered on admission included a complete blood count (CBC) that revealed a neutrophil count of 1,220 cells per microliter (1.22 k/*μ*L). A gastrointestinal (GI) panel of stool revealed the presence of both EPEC and astrovirus nucleic acid. After consultation with the hospital infectious disease service, ciprofloxacin was changed to a 3-day course of azithromycin. The watery diarrhea persisted, but the patient reported marked subjective improvement after completion of the azithromycin course. The patient was ultimately discharged stable and afebrile on loperamide for control of her residual diarrhea.

### 2.2. Case 2

The second patient was a 61-year-old male who was admitted to the hospital for induction chemotherapy following a recent diagnosis of acute myeloid leukemia (AML). A CBC from hospital day 4 revealed a neutrophil count of 1.21 k/*μ*L. The next day, hospital day 5, he developed watery diarrhea. A physical exam revealed no abnormalities. A GI panel of stool revealed EPEC. A 3-day course of azithromycin was initiated. The diarrhea resolved, and the patient finished an additional round of chemotherapy for his AML. The patient's hospital course was complicated by mucositis and pancytopenia with subsequent neutropenic fever, but he was eventually discharged stable, afebrile, and diarrhea-free after 44 hospital days.

### 2.3. Case 3

The third patient was a 70-year-old male with a history significant for duodenal gastrinoma (with Zollinger–Ellison syndrome) who presented for evaluation due to 6 months of bouts of watery diarrhea. He was originally diagnosed with a duodenal gastrinoma over 20 years prior to his presentation at our facility. He was treated at that time with surgical resection of the tumor. At presentation, physical examination revealed a patient in no apparent distress with a soft, nontender, and nondistended abdomen. The patient was found to have an elevated serum gastrin level, and a workup for gastrinoma recurrence was performed. Upper endoscopic series demonstrated erosive esophagitis and a small duodenal polyp. Biopsy of the duodenal polyp revealed a well-differentiated neuroendocrine tumor that was determined to be a gastrinoma. The stool GI panel revealed EPEC, and ciprofloxacin was began. The patient reported resolution of his diarrhea several days after initiating ciprofloxacin and underwent successful surgical removal of the duodenal gastrinoma.

## 3. Discussion

The management of infections caused by *E. coli* can be challenging because of the extensive and varied drug resistance reported for this organism. It is well established that azithromycin has excellent activity against enterotoxigenic *E. coli* (ETEC) and enteroaggregative *E. coli* (EAEC), but limited clinical data supporting the use of azithromycin against EPEC exist [4, 5]. Current guidelines recommend either trimethoprim/sulfamethoxazole, norfloxacin, or ciprofloxacin for definitive antibiotic therapy of EPEC diarrhea in adults [3]. However, data acquired from *in vitro* studies found that azithromycin had a similar minimum inhibitory concentration (MIC) against EPEC as it did against ETEC, enteroinvasive *E. coli* (EIEC), enterohemorrhagic *E. coli* (EHEC), *Salmonella* spp., and *Shigella* spp. [6]. Additionally, there are troubling national trends of increasing fluoroquinolone resistance, particularly among *E. coli* sequence type 131 (ST131) in the general inpatient hospital population [7, 8]. More specifically, upwards of 80% of hospitalized patients with hematologic malignancies receive prophylactic fluoroquinolone therapy [9]. For this reason, cancer patients, especially those with hematologic malignancies, are more likely to have been exposed to fluoroquinolones and thus be colonized or infected with fluoroquinolone-resistant *E. coli*. Therefore, azithromycin may hold special utility for EPEC diarrhea arising in patients with a recent exposure to fluoroquinolones.

Azithromycin has additionally been shown to reduce fecal bacterial shedding during diarrheal episodes with enteroaggregative *E. coli* (EAEC) [10]. It is plausible that this property of reduced bacterial shedding may extend to EPEC as well. This may be significant from an institutional perspective to prevent the dissemination of EPEC diarrhea amongst patients on the same inpatient ward.

A discussion of bacterial diarrhea would not be complete without discussing the recent proliferation of comprehensive GI “panels” that are capable of detecting a wide array of organisms previously unidentifiable in the clinical setting. While these tools have indisputable clinical utility, questions have been raised in cases where these panels return positive for multiple pathogens [11, 12]. Because these assays currently lack quantification, it is often unclear if treatment is indicated in cases where multiple pathogens are detected simultaneously. Further, the proliferation of such culture-independent methods may dampen public health efforts to track outbreaks of diarrheal illness and determine antimicrobial susceptibility [13].

In conclusion, we report 3 cases of EPEC diarrhea in cancer patients. In both case 1 and case 2, azithromycin provided effective relief of diarrheal symptoms. Of note, we believe that our first patient did not experience complete resolution of her diarrhea because of the astrovirus coinfection. In case 3, ciprofloxacin was used (as per current recommendations), and the patient did experience symptomatic relief. Ultimately, azithromycin should be considered as a reasonable alternative to the fluoroquinolones in the treatment of non-*Clostridium difficile* watery diarrhea in adult cancer patients, especially in scenarios where the patient has had recent exposure to fluoroquinolones.
